# Predictors of Outcome of Rectovaginal Fistula Surgery in Women With Crohn’s Disease

**DOI:** 10.14740/jocmr2421w

**Published:** 2015-12-28

**Authors:** Ashish Manne, Malik B. Ahmed, Talha A. Malik

**Affiliations:** a Department of Medicine-Gastroenterology, University of Alabama at Birmingham, Birmingham, AL, USA; b Department of Epidemiology, University of Alabama at Birmingham, Birmingham, AL, USA

**Keywords:** Predictors, Outcome, Crohn’s disease, Perianal fistula, Rectovaginal fistula

## Abstract

**Background:**

Despite measures taken to control traditional risk factors like increased rectal disease activity, anorectal stenosis, and, to a lesser extent, obesity, rates of poor outcomes after rectovaginal fistula (RVF) surgery in women with Crohn’s disease (CD) are still high and require further elucidation. To bridge the gap, we sought to identify risk factors beyond the aforementioned determinants that may be associated with poor outcomes in these patients.

**Methods:**

We designed a retrospective, unmatched, case-control study to test our hypothesis. The population comprised women with CD who underwent RVF surgery between the years of 2000 and 2013. Cases were defined as surgeries with an unsuccessful outcome at 4 weeks post-surgery, and controls represented those with a successful outcome at 4 weeks post-surgery. With multivariable logistic regression models, we sought to identify pertinent risk factors for poor outcomes after RVF surgery in these women.

**Results:**

Of the 108 women with CD who were referred to our institution for evaluation of RVFs between 2000 and 2013, 39 underwent a total of 63 surgeries, of which 16 were cases and 47 were controls. There were no significant differences between them with regard to medications and clinical or mucosal disease severity, but a significantly higher proportion of poor outcomes arose from the group of women who underwent a mucosal flap procedure (88%) compared to those who had a seton placed (13%; P = 0.0004). The final adjusted logistic regression model demonstrated that women who underwent a mucosal flap procedure instead of a seton placement were 17.5 times more likely to have a poor surgical outcome (odds ratio (OR): 17.51; 95% confidence interval (CI): 3.12 - 98.43; P = 0.0012). Moreover, it was seen that women with active colonic mucosal disease, independent of rectal disease activity, were 4.4 times more likely to have a poor outcome (OR: 4.40; 95% CI: 1.06 - 18.26).

**Conclusion:**

Mucosal flap procedures, representing the second, or definitive, stage in surgical treatment of RVF are associated with much worse outcomes and therefore should be preceded by aggressive medical optimization of the patient.

## Introduction

Crohn’s disease (CD), a heterogeneous chronic inflammatory condition, primarily of the gastrointestinal tract, afflicts roughly 750,000 people in the US. It is characterized by transmural inflammation and can involve any part of the tract, from the mouth to the anus. Beyond the typical intestinal manifestations, CD is frequently complicated by extra-intestinal disease, with associated conditions such as uveitis, affecting the eye, ankylosing spondylitis, a type of arthritis, and erythema nodosum, affecting the skin, among others, significantly reducing the quality of life of affected patients. Furthermore, immune modulators, a mainstay of CD treatment, are themselves a harbinger of numerous medical complications. Unfortunately, despite well-characterized genotypic and phenotypic characteristics of affected patients, CD responds variably and unpredictably to treatment [1-3].

Perianal fistulas are common complications of CD, and among Crohn’s associated perianal fistulas, rectovaginal fistulas (RVFs) are a particularly debilitating condition. Recent studies underline inflammatory bowel disease (IBD) as the most common etiology of RVF, with CD responsible for 36% of cases. The literature highlights the propensity for CD-related RVF to recur, and more troubling still, surgical intervention in CD patients with recurrent RVF fails 50% of the time, compared with a 9% failure rate in patients suffering from RVF of non-CD origin [1].

Complicating matters further, Zhu et al found that, among established surgical repair techniques, mucosal advancement flaps were found to have the lowest success rate, at an abysmal and discouraging 29%, yet this surgical intervention often represents mainstream RVF repair, especially in complicated cases, such as those in CD patients. Furthermore, suture dehiscence, a feared complication, occurred in 43% of advancement flap procedures, higher than in other studied surgical methods [2].

A unifying theme among studies detailing successes and failures of RVF intervention is the importance of medical management. Proper initial therapy is considered to be immunomodulator use; most importantly an anti-tumor necrosis factor-alpha (TNF) blocker. TNF blockers are credited with increasing surgical success rates, decreasing rate of recurrence, increased longevity of repair, and overall increased healing [3-6]. According to Valente et al, surgery should not even appear on a clinician’s radar before exhaustive medical therapy has failed beyond a doubt. Often sepsis control and seton placement stand in for definitive surgery [7]. Though the exact details remain murky, what remains clear is that proper immunomodulator use in these patients prior to surgery, representative of a trend toward overall medical optimization, yields the most promising results.

Increased rectal disease activity, anorectal stenosis, and, to a lesser extent, obesity, are well-established determinants of poor outcome. Despite measures taken to control these risk factors, rates of poor outcome after RVF surgery in women with CD remain high and require further investigation. We sought to identify other risk factors that may be associated with poor outcome in these patients.

## Materials and Methods

### Study design, patient population, and selection criteria

Following approval by the University of Alabama’s Office of the Institutional Review Board, we conducted a retrospective unmatched case-control study at University of Alabama Birmingham, at Birmingham, in order to study the various factors affecting outcomes of RVF surgery in complicated CD. Utilizing billing search engines, women with complicated CD who underwent flap surgery or seton placement between the years of 2000 and 2013 were identified.

Subjects were included in the study only if the following data were available on them: age, age at diagnosis of disease, ethnicity, BMI, medication history, duration of disease from date of diagnosis, clinical and mucosal disease activity at the time of surgery, history of RVF surgery, age at surgery, type of surgery, and smoking status.

### Data

Relevant data required for our study were obtained from electronic medical records. Outcome was termed as poor if the patient had a complication (such as wound infections, delayed wound healing, wound dehiscence, or prolonged recovery time during their post-op period or during first post-op) requiring a non-standard intervention either during the post-op recovery period or at the first post-op visit, whether scheduled or unscheduled, such patients were taken as cases for our study, while those who had uncomplicated postoperative care were taken as controls.

Using BMI (calculated using the standard formula: weight in kilograms/square of height in meters), subjects were divided into obese (BMI > 30) and non-obese (BMI between 18.5 and 29.9). Based on guidelines provided by the American College of Gastroenterology, subjects were categorized by disease activity, including: in remission, mild-to-moderate disease, and moderate-to-severe disease.

Subjects were considered to have a history of steroid use if they used conventional oral or parenteral steroids for more than 1 week, rectal or topical steroids for more than 1 month, or oral budesonide for more than 3 months in past 1 year, while usage of steroids in any form within 1 month of surgery was considered as recent steroid use. If subjects were on any biological agent such as azathioprine (AZA), 6-mercaptopurine (6-MP), or methotrexate (MTX) at the time of surgery and on post-op follow-up, they were considered to be on an immune modulator. Subjects smoking at the time of surgery were considered smokers.

### Statistical analysis

Chi-square or Fisher exact test for categorical variables and independent *t*-test for continuous variables (Wilcoxon rank sum test was used when data did not follow normal distribution) were used to compare cases and controls.

To understand the influence of various factors on surgical outcome in RVF surgery in complicated CD, unadjusted odds ratio (OR) and 95% confidence interval (CI) were estimated using logistic regression. In adjusted models, age, ethnicity, duration of disease, and duration of observation were used as covariates.

Statistical tests were two-sided with 5% significance level ( i.e., α = 0.05). Statistical software (SAS, version 9.2; SAS institute, Inc., Cary, NC) was used to perform all statistical analyses.

## Results

We identified 108 women with CD complicated by RVF who were managed at UAB between the years of 2000 and 2013, among whom 39 subjects had RVF-related surgery (either mucosal flap surgery or seton placement). A total of 63 surgeries were performed in these 39 subjects, according to our medical records.

Using the criteria above, we identified 16 surgeries with negative outcomes, termed cases, and 47 surgeries with positive outcomes, termed controls. Comparison between cases and controls did not show major differences with regard to several variables tested as evident in Table 1.

**Table 1:** Comparing Various Characteristics Between Cases and Controls in This Study

Characteristic	Cases (N = 16)	Controls (N = 47)	P-value
BMI, median (range)	24.9 (18.6 - 36.6)	23.6 (18 - 70)	0.502*
BMI categorical			0.718
Obese	3 (21%)	11 (25%)	
Overweight	4 (29%)	8 (18%)	
Standard weight	7 (50%)	25 (56%)	
Type of surgery			0.0004*
Mucosal flap procedure	88%	12%	
Seton placement	13%	77%	
Age at surgery, median (range)	36 (26 - 60)	38 (18 - 78)	0.847
Age at diagnosis, median (range)	28 (20 - 59)	28 (12-78)	0.908
DOD, median (range)	6 (1 - 9)	4 (1 - 110)	0.906
Ethnicity, African American	12 (75%)	26 (55%)	0.165
Past RVF surgery	8 (50%)	20 (43%)	0.759
Smoking	0 (0%)	7 (15%)	0.101
Steroid use	4 (25%)	10 (21%)	0.739
AZA/6-MP use	1 (6.3%)	7 (15%)	0.667 (Fisher)
Biologic use	7 (43%)	29 (61%)	0.212
Methotrexate use	1 (6%)	3 (6%)	1.0 (Fisher)
EIM	2 (13%)	4 (9%)	0.626 (Fisher)
Active clinical disease	8 (50%)	16 (34%)	0.261
Active colonic mucosal disease	9 (56%)	16 (34%)	0.119
Active rectal disease	4 (25%)	15 (32%)	0.223

*Satterthwaite method of comparison.

One major difference between both groups was seen in regards to type of surgery. Among cases, 88% had mucosal flap surgery while only 13% had seton placement while among controls only 1% had mucosal flap surgery and 77% had seton placement. Further, adjusted logistic regression model demonstrated that women who underwent a mucosal flap procedure instead of a seton placement were 17.5 times more likely to have a poor surgical outcome (OR: 17.51; 95% CI: 3.12 - 98.43; P = 0.0012). The risk of poor surgical outcome was also found to be higher in women with active colonic mucosal disease independent of rectal disease (OR: 4.40; 95% CI: 1.06 - 18.26).

## Discussion

RVF accounts for significant and distressing complications in women with CD, interfering with basic human actions, namely sexual activity. Understandably, limitations in this area of life provide a source of endless worry for women afflicted with this unfortunate complication, and we hope to contribute to the growing body of evidence pointing toward a more nuanced approach to RVF treatment in lieu of hasty surgical intervention. Knowledge, backed by good evidence-based medicine will help us choose an effective approach in managing a patient, allowing us to not only reduce the cost of management but also save the patient from undue mental and physical agony brought about by futile treatments.

Though relatively small, this retrospective case-control study granted us interesting insights into RVF management in complicated CD, underscoring the value of choosing the correct surgical intervention and favoring medical therapy before subjecting patients to the knife. We found that age, age of diagnosis, duration of disease, type of medicines used, BMI and even recurrent surgeries do not convincingly correlate with negative outcomes, while type of surgery plays a major role in outcomes, with patients undergoing mucosal flap surgery 17.5 times more likely to experience a negative outcome when compared with patients subjected to seton placement. Predictably, we also found that those with advanced disease at the time of surgery are more prone to bad outcomes than those with early stages of disease, further solidifying the case for aggressive and early medical treatment, a point endorsed by a plethora of other studies.

It is important to note that the medicine the patient was on at the time of surgery or earlier in the course of the disease, i.e. AZA/6-MP versus MTX versus biologic use did not matter, according to this study. Unless the disease is well under control at the time of surgery, the specific medical therapy seems irrelevant, giving weight to the oft-repeated point that optimal medical management is key in managing CD-associated RVFs. Furthermore, RVFs associated with advanced CD are conventionally managed by advanced flap surgery rather than with simple seton placement, further explaining the poor associated outcomes.

Our study adds to the growing concern in the literature that surgery, specifically mucosal flap advancement, is perhaps preemptively performed and highlights the importance of maximum medical management in CD complicated with RVF. We advise against surgical management unless the medical management is enough to keep disease activity at the mild-moderate stage, and if urgent surgery is warranted, we strongly recommend seton placement over and prior to considering a flap.

## Abbreviations

CD: Crohn’s disease
RVF: rectovaginal fistula
TNF-alpha: tumor necrosis factor alpha
AZA: azathioprine
6-MP: 6-mercaptopurine
MTX: methotrexate
SAS: Statistical Analysis Software
